# Review of carcinogenicity of hexavalent chrome and proposal of revising approval standards for an occupational cancers in Korea

**DOI:** 10.1186/s40557-018-0215-2

**Published:** 2018-01-31

**Authors:** Jungwon Kim, Sangyun Seo, Yangho Kim, Dae Hwan Kim

**Affiliations:** 1 Department of Occupational and Environmental Medicine, Kosin University College of Medicine, Busan, Republic of Korea; 2 Department of Occupational and Environmental Medicine, Ulsan University Hospital, University of Ulsan College of Medicine, Ulsan, Republic of Korea; 3 Department of Occupational and Environmental Medicine, Haeundae Paik Hospital, Inje University, Busan, Republic of Korea; 4 Department of Occupational and Environmental Medicine, Kosin University Gospel Hospital, 34 Amnam-dong, Seo-gu, Busan, 602-702 Republic of Korea

**Keywords:** Hexavalent chromium, Occupational exposure, Cancer, Lung neoplasm

## Abstract

**Background:**

The objective of this study is to suggest revised recognition standards for occupational disease due to chromium (VI) by reflecting recent domestic and international research works and considering domestic exposure status with respect to target organs, exposure period, and cumulative exposure dose in relation to the chromium (VI)-induced occupational disease compensation.

**Methods:**

In this study, the reports published by major international institutions such as World Health Organization (WHO) International Agency for Research on Cancer (IARC) (2012), Occupational Safety and Health Administration (OSHA) (2006), National Institute for Occupational Safety and Health (NIOSH) (2013), American Conference of Governmental Industrial Hygienists (ACGIH) (2004), National Toxicology Program (NTP) (2014), and Agency for Toxic Substances and Disease Registry (ASTDR) (2012) were reviewed and the recent research works searched by PubMed were summarized.

**Results:**

Considering the recent research works and the domestic situation, only lung cancer is conserved in the legislative bill in relation to chromium (VI), and the exposure period is not included in the bill. Nasal and paranasal sinus cancer was excluded from the list of cancers that are compensated as the chromium (VI)- induced occupational disease, while lung cancer remains in the list. In the view of legislative unity, considering the fact that only the cancers having sufficient evidence are included in the conventional list of cancers compensated as occupational disease, nasal and paranasal sinus cancer having limited evidence were excluded from the list.

The exposure period was also removed from the legislative bill due to the insufficient evidence. Recent advices in connection with cumulative exposure dose were proposed, and other considerable points were provided with respect to individual occupational relevance.

**Conclusions:**

It is suggested that the current recognition standard which is “Lung cancer or nasal and paranasal sinus cancer caused by exposure to chromium (VI) or compounds thereof (exposure for two years or longer), or nickel compounds” should be changed to “Lung cancer caused by exposure to chromium (VI) or compounds thereof, and lung cancer or nasal and paranasal sinus cancer caused by exposure to nickel compounds”.

## Background

Chromium (VI), which is the subject of this study, is scarce in the nature and mainly produced in industrial sites. Chromium (VI) is a strong oxidants which is highly corrosive and carcinogenic [1–3].

Recent reports including National Institute for Occupational Safety and Health (NIOSH) have reflected recent research findings in the areas of epidemiology, risk assessment, and toxicology on the basis of various carcinogenic researches works [4], while the domestic standards for the recognition of occupational cancer do not reflect these new research works. The Article 37 of the Industrial Accident Compensation Insurance Act and the Article 34, the Paragraph 3, and the Enforcement Decree of the Industrial Accident Compensation Insurance Act and (Amended on June 30, 2014) provide following recognition standards for chromium (VI)-induced occupational cancers compensation.

### 20. Occupatilegional Cancers

*B. “Lung cancer or nasal and paranasal sinus cancer caused by exposure to chromium (VI) or compounds thereof (exposure for two years or longer), or nickel compounds”*

It is necessary to reflect recent domestic and international scientific research works and consider domestic exposure status to revise the recognition standards for occupational cancers due to chromium (VI) by ① verifying relevant cancers including lung cancer and nasal and paranasal sinus cancer, ② confirming clear evidence for the current exposure period condition of “two years of longer,” ③ resetting standards for replaceable information such as cumulative exposure, and ④ reviewing latent period.

## Methods

In this study, the reports prepared by major international institutions such as World Health Organization (WHO) International Agency for Research on Cancer (IARC) (2012), Occupational Safety and Health Administration (OSHA) (2006), NIOSH (2013), American Conference of Governmental Industrial Hygienists (ACGIH) (2004), (NTP) (2014), and Agency for Toxic Substances and Disease Registry (ASTDR) (2012) were reviewed, and recent the research works searched by PubMed for the research literature of the year 2012 or later were summarized.

## Results

### Domestic exposure status

① Domestic distribution and used amount.

Occupational exposure mostly happens through airborne exposure or dermal exposure. Since most of the absorbed Chromium (VI) is reduced, body chromium is mostly Chromium (III). Chromium may be found in most of tissues, but the main repository is the spleen, liver, and bone marrow [5]. According to the work environment status investigation report, the number of workplaces handling Chromium (VI) was 1412, and the total amount was 9084 tons. Domestically distributed Chromium and Chromium (VI) are mostly imported from other countries. In 2012–2015, the import of Chromium (VI)-related materials was 16,909 tons, and the export was 2503 tons, indicating that 5636 tons was imported and 834 tons was exported each year in average [6].

② Major Exposed Work Type and Worker Health Examination Status.

With regard to a survey on the distribution and usage of Chrome reported by KOSHA, the major exposed work types were in the order of manufacturing and metal treatment (45%) > basic compounds production (15%) > special purpose machinery manufacturing (6%) [7]. According to the worker health examination status report by KOSHA, the number of workers handling chromium was 116,960 in Korea [8].

③ Domestic Exposure Level.

In 1993, Park et al. [9] reported that the average concentration of Chromium (VI) exposed to individual workers in plating process was 1.7 μg/m^3^. And Shin et al. [10] investigated the Chromium (VI) exposure to arc welding workers in domestic ship building industry, and reported that the average Chromium (VI) exposure concentration was 20 μg/m^3^ during mild steel welding process and 130 μg/m^3^ during stainless steel welding process. According to a survey on the distribution and usage of Chrome reported by KOSHA, the average chromium (VI) exposure concentration was 2.3 μg/m^3^ during painting process, 1.5 μg/m^3^ during plating process, and 2.0 μg/m^3^ during mixing process in the metal heat treatment plating and other treatment industries, and other related product manufacturing industries [7]. The chromium (VI) exposure assessment performed in individual processes of plating industry revealed that the chromium (VI) concentration was the highest in the plating work (GM = 4.15 μg/m^3^), followed by polishing work (GM = 1.86 μg/m^3^) and other works (GM = 1.28 μg/m^3^) [11].

### Review of carcinogenicity

Among major international institutions, the carcinogenicity of chromium (VI) is intrinsically same. Both of water-soluble and water-insoluble chromium (VI) are classified as group 1(the agent mixture is carcinogenic to humans) by IARC, group K(known to be human carcinogen) by NTP, group A(human carcinogen) by EPA, A1(confirmed human carcinogen) by ACGIH [2, 3, 12, 13]. The summary of carcinogenicity of each organs is as follows.

#### Target organs

① Lung Cancer.

Large-scale cohort studies and many other studies have reported excess risk of lung cancer in workers exposed to chromium (VI). In conclusion, IARC reported that there is sufficient evidence in humans that chromium (VI) causes lung cancer [2]. OSHA reviewed literature about carcinogenic effects in different work types as follows. In chromate production, an increase of lung cancer mortality was identified and a significant increase was found particularly in response to the cumulative exposure dose and the exposure period. In the chromium pigment production, an increase of lung cancer incidents in comparison with standard population was reported, and the risk of lung cancer was higher either in water-soluble chromium or water-insoluble chromium. In chromium plating process where workers are exposed to chromic trioxide (CrO_3_), an increase of lung cancer mortality was verified, although the number of studies analyzed was less than the studies about chromate or chrome pigment production. Excess lung cancer mortality was also found in stainless steel welding workers, but the finding is limited in that welding workers are simultaneously affected by asbestos, nickel, and smoking besides chromium [14]. NIOSH concluded that several studies have provided sufficient data that can verify the quantitative relationship between chromium (VI) and lung cancer and proposed cumulative exposure standards by performing quantitative tests using various models [4]. In conclusion, most of international institutions currently recognize the relationship between chromium (VI) and lung cancer and concluded that there is sufficient evidence that chromium (VI) is related with lung cancer.

② Nose and nasal sinus cancer.

Most of cohort studies performed with respect to works exposed to chromium (VI) did not report relationship between chromium (VI) and nose and nasal sinus cancer. Insufficient number of incidents was main reason for lack of the conclusion. Only three case-control studies were identified. Among them, two case-control studies verified the life time excess risk of nose cancer in workers exposed to chromium (VI) [15, 16]. However, Luce et al. [17] who had the best assessment protocol with respect to exposure did not show the life time excess risk of nose cancer in workers exposed to chromium (VI). Cogliano et al. [2] summarized the IARC report with respect to individual organs and concluded that there is limited evidence in humans that chromium (VI) is related with nose and nasal sinus cancer.

③ Other Cancers.

The carcinogenicity of chromium (VI) in other respiratory organs and digestive organs was found to be less possible or of insufficient evidence [1–3, 14]. However, Welling et al. [18] carried out a meta-study of 56 independent reports with respect to the carcinogenicity of chromium (VI) concluded that chromium (VI) is a suggestive cause of stomach cancer, indicating the need for continued future study about lung cancer risk of chromium (VI).

2) Exposure Period

Studies about the exposure period are contradictory with each other. Although a cohort study verified the relationship with the exposure period, more recent cohort studies did not verify a significant relationship with the exposure period, only suggesting a relationship with cumulative exposure [19, 20]. Hence, it is recommended that the current regulation about the exposure period should be eliminated, and referring to cumulative exposure dose rather than exposure period seems reasonable.

3) Latent Period

With respect to latent period, it was considered to provide an estimated latent period to be 10 years according to the latent period of general solid cancers or lung cancer, and five years in the case of high exposure concentration. However, since the variation among studies is large, it is difficult to make a conclusion about the latent period [19, 21].

4) Cumulative Exposure

With respect to cumulative exposure, following results was mainly limited to lung cancer because lung cancer has relatively abundant data and basis in comparison with cancers of other organs such as nasal, gastrointestinal cancers. NIOSH study which was based on ‘Baltimore Cohort’ and ‘Painesville Cohort’ that had abundant exposure information in comparison with other previous cohort studies reported that in the case of 45 years of exposure at the level of 1 μg/m^3^-yr, which is the previous NIOSH recommended exposure limit (REL), 6 out of 1000 lifetime excess risk of lung cancer death had noted, and in the case of 45 years of exposure at the level of 0.2 μg/m^3^-yr, 1 out of 1000 lifetime excess risk of lung cancer death had identified [4]. Therefore, NIOSH proposed a new REL (0.2 μg/m^3^-yr), and suggested a cumulative exposure standard of 0.009mg/m^3^-yr according to it. The validity of the NIOSH study is based on that the most extensive quantitative exposure data was used and the results were similar to those of previous other reports [1, 3, 14]. Apart from NIOSH, Seidler et al. [22] performed meta-study to set occupational exposure standards in Germany and concluded that excess lung cancer risk was found in 0.03 out of 1000 at the cumulative lifetime exposure level of 0.004mg/m^3^-yr and in 3.36 out of 1000 at the cumulative lifetime exposure level of 0.04mg/m^3^-yr, based on two cohort studies [23, 24]. In Germany, the exposure standards based on cumulative exposure have three levels by German Committee on Hazardous Substances: ① acceptable: excess risk found in 0.4 or less out of 1000, ② tolerable: excess risk found in 0.4 to 4 out of 1000, and ③ not tolerable: excess risk found in 4 or more out of 1000 [25]. Applying the above criteria to the study results by Seidler et al. [22], the tolerable exposure level is estimated to be less than 0.04mg/m^3^-yr, and the acceptable exposure level less than 0.004mg/m^3^-yr. In addition, racial difference in the sensitivity, which is important in extrapolating overseas study results to domestic population, was not found. Therefore, the cumulative exposure (0.009 mg/m^3^-yr) may be considered also in Korea to assess the causal relation.

5) Ratio of chromium (VI) to total chromium

Previously, total chromium, not chromium (VI), was measured in a number of working environment investigation. In such cases, chromium (VI) exposure should be evaluated indirectly, which may be done by referring to domestic and international reports about the ratio of chromium (VI) to total chromium in each work type. Shin et al. [10] showed that the average ratio of chromium (VI) to total chromium was 35.5% in the MIG-mild steel welding, while it was 8.4%(6.3–9.7%) in the MIG-stainless steel welding. Lee et al. [11] reported that the average ratio of chromium (VI) to total chromium was 31% in the plating worker group.

6) Other Considerations

Although insufficient epidemiological study has been performed yet with respect to various water-insoluble compounds and water-soluble compounds, it is concluded that the cumulative exposure assessment should be performed regardless of the forms of chromium (VI) compounds, because the animal study result on water-insoluble compounds and water-soluble compounds showed that the cancer risk of water-insoluble chromium (VI) compounds is similar to that of water-soluble chromium (VI) compounds [26].

Other considerations are as follows. Since the certain industries, such as chromic acid production, plating, and paint pigment production have relatively clear evidence of lung cancer carcinogenicity, they can be referred when individual case is evaluated. According to Gibb et al. [21], mucosal irritation signs (nasal septum irritation, nasal septum ulcer, nasal septum perforation, skin ulcer, skin irritation, skin inflammation, burn, and keratitis) may indicate high-concentration exposure, and thus such symptoms should be taken into consideration. This is based on the fact that irritation symptoms themselves are indicators of exposure and that inflammations may trigger oncogenesis [21, 27].

## Discussion

Considering the recent studies and the domestic status, it is suggested that the current recognition standard which is “Lung cancer or nose and nasal sinus cancer caused by exposure to chromium (VI) or compounds thereof (exposure for two years or longer), or nickel compounds” should be changed to “Lung cancer caused by exposure to chromium (VI) or compounds thereof, and lung cancer or nasal and paranasal sinus cancer caused by exposure to nickel compounds”.

First of all, nasal and paranasal sinus cancer was excluded from the list of cancers that are compensated as the chromium (VI)-induced occupational cancers, while lung cancer remains in the list. This is because IARC also classifies nasal and paranasal sinus cancer as having limited evidence in human. Of course, such classification does not contradict the nasal and paranasal sinus cancer carcinogenicity. However, considering the fact that the conventional list of cancers compensated as occupational cancers includes only cancers having sufficient evidence, this revision is for maintaining legislative unity rather than contradicting the carcinogenicity. Other organs including stomach and other digestive organs showed low carcinogenicity or had insufficient evidence.

It was reasonably determined that the exposure period or latent period needed to be removed from recognition standards due to the insufficient academic evidence, even though France and Bulgaria provide recognition standards. However, it was concluded that individual cases should be separately assessed by considering the general sold tumor latent period of 10 years as well as exposure intensity and circumstances. On the other hand, lifetime cumulative exposure to chromium (VI) may be used for the assessment of individual occupational relevance. The lifetime cumulative exposure higher than 0.009mg/m^3^-yr, which is the level suggested by NIOSH (2013), may be considered as being highly related. In previous domestic exposure assessment, total chromium level was often measured. More accurate assessment may be possible if conventional research results on the ratio of chromium (VI) to total chromium are referred to. In addition, information such as mucus irritation signs including nasal septum irritation signs and nasal septum ulcer may be included in the occupational relevance assessment, since the information may imply high concentration exposure.

## Conclusion

Considering the recent studies and the domestic exposure status, the recognition standard is suggested to be *“Lung cancer caused by exposure to chromium (VI) or compounds thereof, and lung cancer or nasal and paranasal sinus cancer caused by exposure to nickel compounds”.*
